# Garlic Polysaccharides Ameliorate AOM/DSS-Induced Colon Tumorigenesis: A Multi-Omics Mechanistic Study

**DOI:** 10.3390/foods15111901

**Published:** 2026-05-28

**Authors:** Yongqiu Qi, Xiaoming Lu, Lingyu Li, Zhenjia Zheng, Yiteng Qiao

**Affiliations:** Key Laboratory of Food Nutrition and Health in Universities of Shandong, College of Food Science and Engineering, Shandong Agricultural University, 61 Daizong Street, Tai’an 271018, China; yongqiuqi@163.com (Y.Q.); lxmwwt@sdau.edu.cn (X.L.); sdnydxlly@163.com (L.L.); zhengzhenjia@sdau.edu.cn (Z.Z.)

**Keywords:** garlic polysaccharides, anti-tumor, anti-inflammatory, colitis-associated colon tumorigenesis, gut microbiota, intestinal metabolites, transcriptomics

## Abstract

As important sources of prebiotics, natural plant polysaccharides have been widely investigated owing to their excellent anti-inflammatory, anti-tumor, immunomodulatory, and gut microbiota-regulating effects. This study explored the anti-tumor effects and underlying mechanisms of garlic polysaccharides (GP-1) in a mouse model of azoxymethane/dextran sulfate sodium-induced colitis-associated colon tumorigenesis. GP-1, identified as a typical fructan (molecular weight: 3583 Da) with (2→1) and (2→6) linkages, significantly improved survival rate, reduced colon tumor burden, and alleviated intestinal bleeding in tumor mice. Furthermore, intestinal damage was significantly attenuated, as evidenced by enhanced barrier integrity, downregulated pro-inflammatory cytokines, and elevated antioxidant enzyme activities. GP-1 also inhibited aberrant epithelial proliferation by suppressing Ki67 protein expression. Multi-omics analyses revealed that these benefits might be associated with gut microbiota and metabolite remodeling, as well as transcriptional suppression of key inflammatory/tumorigenic pathways. Our findings highlighted the inhibitory effect of GP-1 on colon tumorigenesis and supported its potential as a gut-health-promoting functional food ingredient.

## 1. Introduction

Colon tumor is a digestive tract disease closely associated with inflammatory bowel disease, diet, lifestyle, and obesity [1,2]. Chronic inflammation, prevalent in conditions like ulcerative colitis (which confers a 2.4-fold higher risk of tumor development), promotes neoplastic transformation by driving epithelial dysfunction and proliferation [3,4]. Intestinal inflammation and tumorigenesis are influenced by gut microbiota. Elevated levels of enterotoxigenic *Bacteroides fragilis*, *Escherichia coli*, and *Fusobacterium nucleatum* have been linked to colon tumor development [5]. These microbes and their metabolites (e.g., lipopolysaccharide and genotoxins) exacerbate tissue damage and immune dysregulation. Therefore, targeted modulation of gut microbiota and their metabolites represents a promising approach for the prevention and treatment of colon tumors.

Natural polysaccharides are extensively investigated in the biomedical field for their satisfactory bioactivities. For instance, *Ganoderma lucidum* polysaccharide alleviated colon inflammation and tumorigenesis by modulating microbial dysbiosis [6]. In parallel, *Sparassis latifolia* polysaccharides ameliorated metabolic dysregulation in colon tumor mice by selectively enriching probiotics and suppressing pathogenic bacteria [7]. However, most existing studies have focused on the anti-inflammatory and anti-tumor effects of polysaccharides from a single biological perspective, lacking systematic analysis of multiple factors (e.g., gut microbiota, metabolites, and gene expression) in the gut environment. As main active components in garlic, garlic polysaccharides are renowned for their potent anti-inflammatory and prebiotic properties [8]. Our previous research characterized garlic polysaccharides with a backbone of (2→1)-linked *β*-d-Fruf residues with (2→6)-linked *β*-d-Fruf branches [9], which could resist gastrointestinal digestion and alleviate dextran sulfate sodium (DSS)-induced colitis by restoring gut microbiota and barrier integrity [10]. Although garlic polysaccharides possess favorable prebiotic properties, their efficacy and mechanism in colitis-associated colon tumorigenesis remain unclear, particularly regarding their interactions with gut microbiota, metabolites, and intestinal gene expression. We therefore hypothesize that garlic polysaccharides (GP-1) ameliorate azoxymethane (AOM)/DSS-induced colon tumorigenesis by reshaping gut microbiota composition, regulating key metabolic profiles, and inhibiting inflammatory and tumorigenic signaling pathways, thereby alleviating intestinal inflammation, reducing tumor burden, and improving intestinal barrier integrity. The AOM/DSS-induced colitis-associated tumor model closely mimics the progression from inflammation to dysplasia to tumorigenesis in human ulcerative colitis-associated colon tumors.

This research aimed to elucidate the anti-tumor activity and potential mechanism of GP-1 in an AOM/DSS-induced mouse model. GP-1 was isolated from garlic according to established methods and characterized using spectroscopic and chromatographic techniques. The anti-tumor efficacy of GP-1 was systematically investigated through evaluating the disease activity index (DAI), survival rate, tumor burden, intestinal inflammation, and pathological damage in colon tumor mice. Subsequently, multi-omics analysis, incorporating 16S rRNA sequencing, metabolomics, and colon transcriptomics, revealed that GP-1 exerted protective effects on colitis-associated colon tumorigenesis by restoring gut microbiota and metabolic homeostasis, and suppressing inflammatory and tumorigenic pathways. This integrated analysis provides a novel and comprehensive perspective on the anti-tumor mechanism of dietary polysaccharides.

## 2. Materials and Methods

### 2.1. Materials and Reagents

Garlic was purchased from Laiwu (Jinan, China) with no diseases and insect pests. DEAE-52 cellulose was obtained from Yuanye Bio-Technology Co., Ltd. (Shanghai, China). AOM and DSS (molecular weight: 36,000–50,000) were obtained from MP Biomedicals (Irvine, CA, USA). Aspirin was acquired from Sigma-Aldrich (St. Louis, MO, USA). Anti-Muc2, anti-Claudin-1, anti-Occludin, anti-ZO-1 and anti-Ki67 antibodies were procured from Servicebio Technology Co., Ltd. (Wuhan, China).

### 2.2. Preparation of GP-1

Fresh garlic was peeled, sliced, and then dried in a PH070A electrothermal constant-temperature blast drying oven (Shanghai Yiheng Scientific Instrument Co., Ltd., Shanghai, China) at 50 °C for 24 h, followed by crushing. Garlic powder was extracted twice with deionized water (80 °C, 3 h). After vacuum concentration, Sevag reagent (chloroform:*n*-butanol = 4:1, *v*/*v*) was mixed with the extract at a volume ratio of 1:5. The mixture was vigorously shaken and centrifuged to remove the denatured protein layer until no protein precipitation was observed. The polysaccharide solution was subjected to alcohol precipitation and freeze-drying to obtain garlic crude polysaccharides. Subsequently, 5 g crude polysaccharides were dissolved in 80 mL of deionized water and transferred to the DEAE cellulose column. The column was eluted with deionized water at a flow rate of 4 mL/min to obtain the garlic polysaccharides (named GP-1), followed by dialysis and lyophilization. The total carbohydrate content of GP-1 was 98.97%.

### 2.3. Structural Characterization

The molecular weight (Mw) of GP-1 was evaluated using high-performance liquid chromatography (Shimadzu, Kyoto, Japan) coupled with Shodex OHpak SB-8.2 and -802.5 gel columns. The Fourier transform infrared (FTIR) spectrum of GP-1 was scanned using a Nicolet IS10 spectrometer (Thermo Fisher, Waltham, MA, USA) [9]. The ultraviolet–visible (UV–Vis) spectrum was collected using a UV-2600i spectrophotometer (Shimadzu, Kyoto, Japan). The monosaccharide composition was detected by high-performance anion-exchange chromatography (Thermo Fisher, Waltham, MA, USA), following the method of Qiu et al. [10].

### 2.4. Animal Procedures

**Figure 1:**
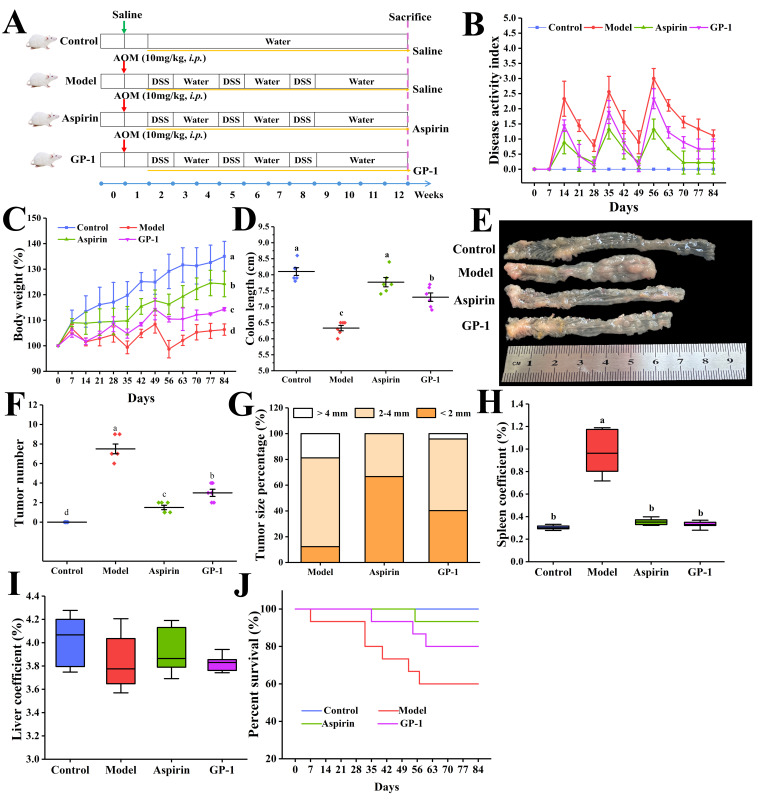
Effect of GP-1 on colon tumorigenesis in AOM/DSS-treated mice. (**A**) Experimental protocol of AOM/DSS mouse model; (**B**) disease activity index; (**C**) body weight changes; (**D**) average colon length; (**E**) representative photographs of longitudinally dissected colons; (**F**) number of colon tumors; (**G**) tumor size; (**H**) spleen coefficient; (**I**) liver coefficient; (**J**) survival rate. All values are presented as mean ± SD (n = 6). Different letters mean significant differences (*p* < 0.05).

Sixty 7-week-old SPF male ICR mice, purchased from Jinan Pengyue Laboratory Animal Breeding Co., Ltd. (Jinan, China), were housed in a barrier environment with a temperature of 24–26 °C and relative humidity of 50–60%, with five per cage. Only male mice were used in this study to exclude the influence of estrogen on AOM/DSS-induced colon tumorigenesis [11]. After acclimation, mice were randomly divided into four groups (n = 15) using a computer-generated random number sequence. The randomization was performed by an investigator not involved in subsequent animal handling or outcome assessment. The four groups were control, model, aspirin (25 mg/kg body weight), and GP-1 (350 mg/kg body weight). A dose of 350 mg/kg body weight of GP-1 was chosen according to preliminary safety data and previous reports demonstrating the efficacy of similar polysaccharides at comparable doses [12,13]. The sample size was determined by considering the statistical requirements and the potential attrition rate due to model failure or animal death. All animal experiments, including group allocation, treatment administration, daily monitoring, and sample analysis, were performed in a blind manner to minimize observer bias. The colon tumor model was constructed using AOM and DSS as described by Wei et al. [7], and the experimental protocol was shown in Figure 1A.

From day 8, all mice received daily oral administration of 0.2 mL of treatment. Aspirin served as a positive control based on its established role in reducing the risk of colorectal adenoma [14]. DAI was assessed weekly by monitoring weight loss, stool consistency, and intestinal bleeding, with scores calculated as described in Table S1. After 12 weeks, mice were euthanized by cervical dislocation under isoflurane anesthesia, and blood was collected for serum separation. The colon was excised, rinsed, and longitudinally opened for tumor inspection. A portion of the colon was fixed in 4% paraformaldehyde, and the remaining tissue was stored at −80 °C. After rinsing with normal saline, the spleen and liver were blotted dry and weighed. The visceral coefficient was expressed as [organ weight (g)/pre-euthanasia body weight (g)] × 100%. All animal procedures complied with the ARRIVE guidelines, the U.K. Animals (Scientific Procedures) Act 1986, and associated guidelines. This research received approval from the Animal Care and Use Ethics Committee of Shandong Agricultural University (SDAUA-2024-226).

### 2.5. Pathological Observation of Colon

After fixation for 24 h, the colon tissue was dehydrated, embedded, and sectioned with a microtome into 5 μm slices. These specimen sections were subjected to histological staining with hematoxylin and eosin (H&E) and Alcian blue-periodic acid-Schiff (AB-PAS), respectively, and then observed using an Olympus IX73 microscope (Kyoto, Japan) to determine the pathological damage of the colon.

### 2.6. Determination of Inflammatory, Immune, and Antioxidant Indicators

Colon tissues were homogenized with phosphate-buffered saline (1:9, *w*/*v*) and centrifuged. The levels of inflammatory mediators (TNF-α, IL-6, IFN-γ, IL-1β, IL-10, iNOS, COX-2, CXCL1, MCP-1) and immune cell markers (CD68^+^, CD3^+^) in supernatants were detected employing enzyme-linked immunosorbent assay kits (Shanghai Enzyme-linked Biotechnology Co., Ltd., Shanghai, China). Mouse serum was diluted with phosphate-buffered saline to determine catalase (CAT), superoxide dismutase (SOD), myeloperoxidase (MPO), and malondialdehyde (MDA) levels according to the kit instructions.

### 2.7. Immunohistochemistry Staining

As reported by Jayachandran et al. [15], paraffin sections (5 μm) were dewaxed and hydrated with xylene and graded ethanol. After antigen retrieval in citrate buffer, the sections were blocked, incubated with primary antibodies, and then incubated with a secondary antibody. Diaminobenzidine color development solution and hematoxylin were added to the sections, which were then visualized under a light microscope, and the average optical density (AOD) was quantified using ImageJ (version 2.14.0).

### 2.8. Short-Chain Fatty Acids (SCFAs) Analysis

Fecal samples (100 mg) were acidified with 10% sulfuric acid and mixed with 1 mL ethyl acetate. After centrifugation at 10,000 rpm for 10 min, the organic phase was collected, filtered through a 0.22 μm membrane, and then detected using a GC-MS system equipped with a VF-WAXms column. The inlet and electron impact ion source were maintained at 250 °C and 230 °C, with helium as the carrier gas.

### 2.9. 16S rRNA Sequencing

Fecal samples were collected from six mice per group (n = 6) and stored at −80 °C. Genomic DNA was then extracted using the E.Z.N.A.^®^ Stool DNA Kit, and the V3–V4 region of 16S rRNA was amplified with the primers 341F/805R as described by Logue et al. [16]. The amplicons were sequenced on the Illumina NovaSeq PE250 platform (Illumina Inc., San Diego, CA, USA). The sequencing data were processed by QIIME2 for diversity analysis, taxonomically annotated against the SILVA and NT-16S databases, and analyzed for differential abundance using the Kruskal–Wallis test (*p* < 0.05). Linear discriminant analysis effect size (LEfSe, LDA > 3.0, *p <* 0.05) was performed to analyze the metagenomic biomarkers among groups. The sample size (n = 6 per group) was selected with reference to the microbiome study conducted by Qiu et al. [10] to ensure adequate statistical power for the above statistical analyses.

### 2.10. Untargeted Metabolomic Analysis

Metabolites were extracted from colon tissues with 80% methanol and analyzed using a UPLC-Q-Exactive system (Thermo Fisher, Waltham, MA, USA) in both positive and negative ion modes. Full MS scans covered a range of *m*/*z* 70–1050 with a resolution of 70,000. The raw data were converted to mzXML format and processed using the XCMS, CAMERA, and metaX packages in R software (version 3.4.4) for peak picking, peak grouping, retention time correction, second-round peak grouping, and annotation of isotopes/adducts. Ions were identified based on retention time and *m*/*z*, and the peak intensities of metabolites were normalized using median normalization. Metabolites were annotated against the Human Metabolome Database and Kyoto Encyclopedia of Genes and Genomes (KEGG) database. Differential analysis was performed using metaX, while partial least squares discriminant analysis (PLS-DA) and variable importance in projection (VIP) calculation were conducted using the ropls package. Differential metabolites were selected based on *p* < 0.05, VIP ≥ 1, and fold change (FC) ≥ 1.2 or FC ≤ 0.83. KEGG pathway enrichment analysis was performed using the hypergeometric test, with *p* < 0.05 considered statistically significant. The sample size (n = 4 per group) was determined based on the metabolomic study by Du et al. [17] to ensure adequate statistical power for differential metabolite screening and pathway enrichment analysis.

### 2.11. Transcriptome Sequencing

Total RNA was extracted from colon tissues using TRIzol reagent, and high-quality RNA (RIN > 7.0) was utilized to construct sequencing libraries. The paired-end sequencing was run on the Illumina NovaSeq 6000 platform (Illumina Inc., San Diego, CA, USA). Following sequencing, StringTie (version 2.1.6) was employed for transcriptome assembly and gene expression quantification. DESeq2 was used to identify differentially expressed genes (DEGs) with |log_2_FC| ≥ 1 and *p* < 0.05. Gene expression patterns were visualized by principal component analysis (PCA) and cluster heatmaps, and functional enrichment of DEGs was analyzed through Gene Ontology (GO) terms and KEGG pathways. The sample size (n = 4 per group) was selected in line with the transcriptomic design described by Li et al. [18] to ensure adequate statistical power for DEGs identification and functional enrichment analysis.

### 2.12. Statistical Analysis

Experimental data were presented as the mean ± standard deviation (SD). Statistical differences were assessed by one-way ANOVA using IBM SPSS Statistics software (version 28.0), followed by Tukey’s post hoc test for multiple comparisons. *p* < 0.05 was considered statistically significant.

## 3. Results

### 3.1. Structural Characterization of GP-1

In Figure S1A, the Mw distribution of GP-1 showed a single symmetrical peak. Based on the dextran standards, the Mw of GP-1 was 3583 Da, which was nearly identical to the result reported in our previous study (3684 Da), reflecting the excellent reproducibility of extraction and purification methods [9]. The infrared spectrum in Figure S1B exhibited typical signals of fructan, where the absorption peaks in the polysaccharide fingerprint region (1000.0–800.0 cm^−1^) suggested the presence of *β*-configuration glycosidic bonds in GP-1 [19]. As shown in Figure S1C, the absence of absorption peaks (260–280 nm) in the UV–Vis spectrum confirmed the high purity of GP-1 by ruling out the presence of nucleic acids or proteins. As a fructan, GP-1 was primarily composed of fructose and glucose (93.58:6.42, Figure S1D), similar to previously prepared garlic polysaccharides [9]. In this study, GP-1 (obtained from the same source and extraction procedure) showed structural features highly compatible with those of garlic polysaccharides described in previous studies [9]. Therefore, GP-1, featuring a backbone of (2→1)-linked *β*-d-Fruf residues with (2→6)-linked *β*-d-Fruf branches, was replicated in this work.

### 3.2. GP-1 Attenuated AOM/DSS-Induced Colon Tumorigenesis

Prolonged inflammatory exposure was associated with elevated DAI in AOM/DSS-treated mice, accompanied by weight loss, intestinal bleeding, and diarrhea (Figure 1B). GP-1 and aspirin treatments effectively reduced the DAI score. Correspondingly, the marked weight loss in the model group was mitigated by GP-1 (Figure 1C), with the final body weight 7.55% higher than that in model mice (*p* < 0.05). AOM/DSS treatment also induced obvious colon atrophy (6.33 cm in the model group vs. 8.10 cm in the control group), which was partially reversed by the GP-1 administration (7.30 cm, *p* < 0.05) (Figure 1D). To visualize the effect of GP-1 on tumorigenesis, colon tissues were longitudinally dissected as outlined in Figure 1E. Macroscopic observation showed that the AOM/DSS-treated model group developed numerous colon tumors of diverse dimensions, whereas GP-1 intervention was associated with fewer and smaller tumors. Compared with the model group, GP-1 administration reduced tumor number by 60.00% and the proportion of tumors >2 mm by 31.90% (*p* < 0.05) (Figure 1F,G). The spleen coefficient was increased by 219.13% in model mice relative to controls, and was reduced by 65.95% and 63.41% following GP-1 and aspirin administration, respectively, relative to the model group (*p* < 0.05, Figure 1H). No significant difference in liver coefficient was observed among groups (Figure 1I). Together with the improved survival rates (80% in the GP-1 group vs. 60% in the model group) (Figure 1J), these results supported the protective effect of GP-1 against AOM/DSS-induced colon tumorigenesis.

### 3.3. GP-1 Improved Intestinal Mucosal Barrier Integrity in AOM/DSS-Treated Mice

**Figure 2:**
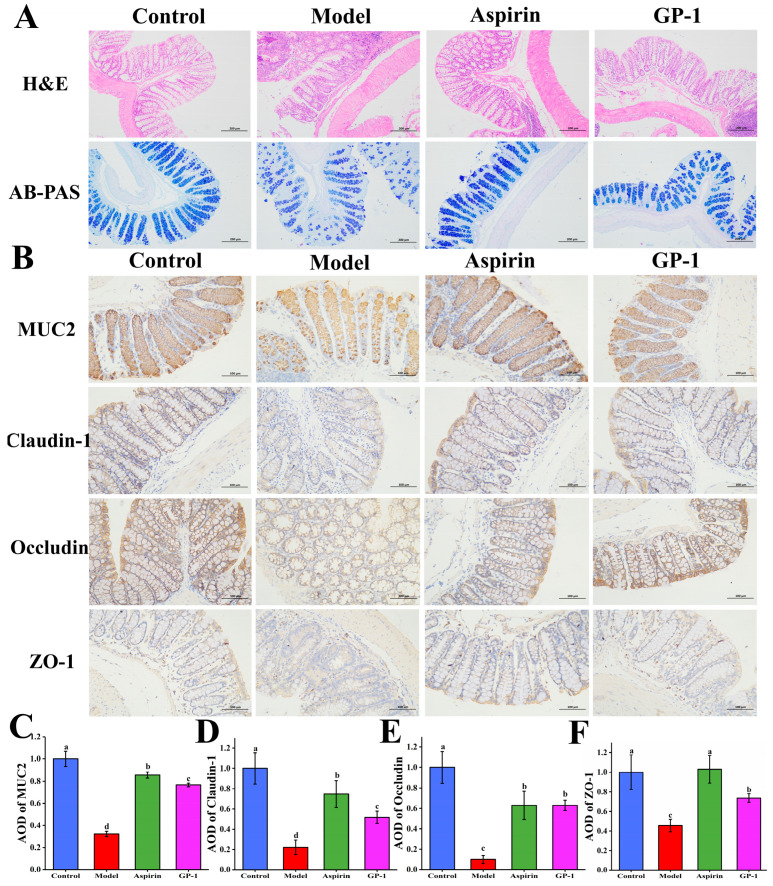
Effect of GP-1 on intestinal barrier. (**A**) Representative pathological images of H&E and AB-PAS staining, 200 μm; (**B**) immunohistochemical staining of MUC2, Claudin-1, Occludin and ZO-1, 100 μm; (**C**–**F**) average optical density of MUC2, Claudin-1, Occludin and ZO-1. All values are presented as mean ± SD (n = 3). Different letters mean significant differences (*p* < 0.05).

Histopathological analysis revealed severe mucosal disruption, crypt deformation, and atypical hyperplasia in model mice (Figure 2A). In comparison, GP-1 and aspirin groups showed relatively intact mucosal architecture and reduced epithelial damage, although some lymphocytes and polymorphonuclear cells persisted in the lamina propria. AB-PAS staining revealed depleted goblet cells and mucus in model mice, a key feature of colon tumorigenesis that was notably reversed by GP-1 (Figure 2A). Furthermore, the expression of MUC2 was analyzed by immunohistochemistry (Figure 2B). AOM/DSS treatment markedly reduced MUC2 expression to 32.35% (AOD) of the control level (*p* < 0.05), and GP-1 intervention restored MUC2 to 76.69% of the control level (Figure 2C).

Tight junctions are critical for the colon epithelial barrier function. In the model group, the expression of Claudin-1, Occludin, and ZO-1 was significantly suppressed (Figure 2B). Instead, GP-1 treatment significantly elevated the levels of these proteins, with AOD values of Claudin-1 and Occludin increased by 1.34-fold and 5.48-fold, and ZO-1 elevated by 61.62% compared with the model group (Figure 2D–F). These observations suggested a potential association between GP-1 and enhanced intestinal barrier function via modulation of mucin and tight junction proteins—a finding consistent with a previous report on *Poria cocos* polysaccharides [20].

### 3.4. GP-1 Suppressed AOM/DSS-Induced Colon Inflammation

**Figure 3:**
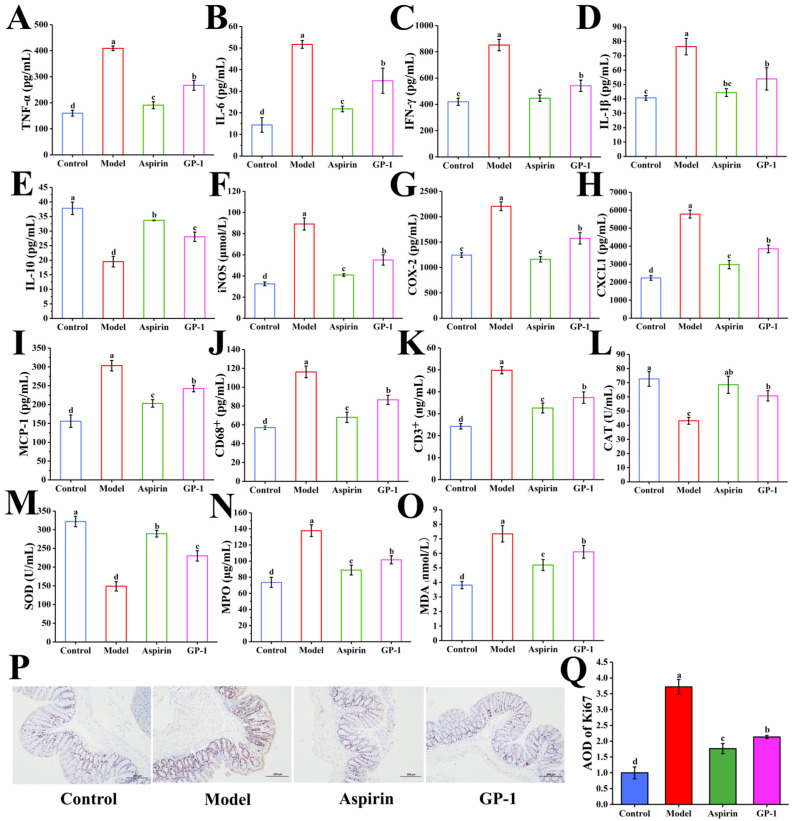
Effects of GP-1 on inflammation, oxidative stress, and cell proliferation. (**A**) TNF-α; (**B**) IL-6; (**C**) IFN-γ; (**D**) IL-1β; (**E**) IL-10; (**F**) iNOS; (**G**) COX-2; (**H**) CXCL1; (**I**) MCP-1; (**J**) CD68^+^; (**K**) CD3^+^; (**L**) CAT; (**M**) SOD; (**N**) MPO; (**O**) MDA; (**P**) immunohistochemical staining of Ki67, 200 μm; (**Q**) average optical density of Ki67. All values are presented as mean ± SD (n = 3–6). Different letters mean significant differences (*p* < 0.05).

As shown in Figure 3A–D, AOM/DSS treatment markedly elevated the levels of pro-inflammatory cytokines (TNF-α, IL-6, IFN-γ, and IL-1β). TNF-α, produced by macrophages and T cells, was significantly elevated in the model group (from 160.22 pg/mL to 409.25 pg/mL, *p* < 0.05) (Figure 3A) [21], and GP-1 intervention resulted in a 34.72% reduction in the colon TNF-α level (*p* < 0.05). IL-6 is an important mediator between inflammation and tumor [22], and its level decreased to 67.47% of the model group following GP-1 treatment (*p* < 0.05, Figure 3B). Excessive IFN-γ is known to impair the mucosal barrier integrity [23], and its concentration was decreased by 36.40% in the GP-1 group compared with the model group (Figure 3C). Simultaneously, GP-1 treatment significantly reduced the IL-1β level by 29.39% relative to the model group, an efficacy comparable to that of aspirin (Figure 3D). Meanwhile, the level of the anti-inflammatory cytokine IL-10 in the GP-1 group was increased by 43.77% compared with the model group (*p* < 0.05) (Figure 3E).

The potential anti-inflammatory properties of GP-1 were further supported by the downregulation of key inflammatory enzymes. AOM/DSS-induced upregulation of iNOS and COX-2 was significantly attenuated by GP-1 intervention, with their levels decreased by 38.16% and 28.61% (Figure 3F,G). Chung et al. [24] also found that *Aster glehni* could inhibit colon tumorigenesis by downregulating iNOS, COX-2, and pro-inflammatory cytokines. Overall, these results indicated that the inhibitory effect of GP-1 on inflammatory mediators might be associated with its alleviation of colitis-associated colon tumorigenesis.

### 3.5. GP-1 Improved Immune Homeostasis in AOM/DSS-Treated Mice

Cytokine dysregulation can initiate intestinal immune responses by recruiting immune cells to lesion sites [25]. CXCL1, a key chemoattractant for macrophages and neutrophils, was increased by 1.59-fold in the model group compared with the normal mice (Figure 3H). GP-1 and aspirin treatments significantly reduced CXCL1 by 33.37% and 48.51%, respectively, relative to the model group (*p* < 0.05). Similarly, MCP-1, which facilitates T-lymphocyte and macrophage infiltration [26], increased by 94.50% in the model group compared with the control group (Figure 3I), and this increase was significantly attenuated by 20.04% after GP-1 administration. Excessive immune cell infiltration is related to the progression of colon tumorigenesis. As shown in Figure 3J,K, AOM/DSS treatment caused marked infiltration of CD68^+^ macrophages and CD3^+^ T cells in the colon, and these increases were partially reversed by GP-1 (reductions of 25.52% and 24.98%, respectively). Taken together, GP-1 treatment could ameliorate AOM/DSS-induced dysregulated immune cell infiltration in the colon, consistent with the findings of Wei et al. [7].

### 3.6. GP-1 Relieved Systemic Oxidative Stress in AOM/DSS-Treated Mice

Inflammatory cell infiltration can trigger oxidative stress, thereby exacerbating DNA damage and inflammation-driven tumorigenesis [27]. Compared with the control group, AOM/DSS treatment significantly decreased the activities of antioxidant enzymes CAT and SOD by 40.72% and 53.79%, respectively (*p* < 0.05) (Figure 3L,M). GP-1 administration relieved these decreases, elevating CAT and SOD activities by 40.94% and 54.59% relative to the model group. Concurrently, GP-1 significantly reduced the activity of MPO (an oxidant-producing enzyme) by 26.27% and lowered the serum MDA level (Figure 3N,O). MDA has been reported to be associated with intestinal adenoma development [28]. These results suggested that GP-1 treatment could alleviate AOM/DSS-induced oxidative damage. Notably, our prior study on garlic polysaccharide-iron complexes also showed enhanced systemic antioxidant capacity in iron deficiency anemia mice [9], further underscoring the potential of garlic polysaccharides as natural antioxidants for maintaining intestinal health.

### 3.7. GP-1 Inhibited Tumor Development in AOM/DSS-Treated Mice

Aberrant cell proliferation is a hallmark of colon tumorigenesis. In normal colon tissue, Ki67 was mainly distributed in the crypt base with low intensity (Figure 3P). In contrast, AOM/DSS treatment profoundly disrupted this homeostasis, leading to a significant intensification and upward expansion of Ki67-positive cells. Compared with the control group, the AOD of Ki67 was increased by 2.72-fold in the model group (*p* < 0.05) (Figure 3Q). Oral administration of GP-1 significantly lowered Ki67 expression, resulting in a 42.62% reduction in AOD relative to the model group. This suppression of proliferative activity aligned with findings reported by Chen et al. [20] and further supported the potential of GP-1 in inhibiting colon tumor progression.

### 3.8. GP-1 Modulated Intestinal Flora Diversity in AOM/DSS-Treated Mice

**Figure 4:**
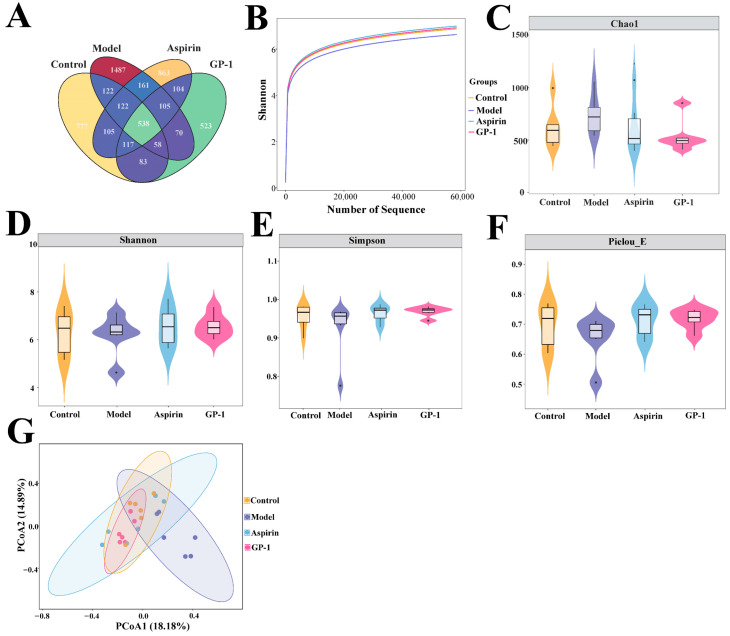
Effect of GP-1 on intestinal flora diversity in AOM/DSS-treated mice. (**A**) Venn diagram of ASVs; (**B**) rarefaction curve; (**C**–**F**) alpha diversity indices; (**G**) beta diversity analysis (n = 6).

To investigate the changes in gut microbiota, 16S rRNA sequencing was conducted on fecal samples from all groups. A total of 5235 amplicon sequence variants (ASVs) were identified, with 538 shared among all groups and unique ASVs of 777, 1487, 863, and 523 in the control, model, aspirin, and GP-1 groups, respectively (Figure 4A). The plateauing rarefaction curves verified sufficient sequencing depth (Figure 4B). Alpha diversity, including Chao1, Pielou_E, Shannon, and Simpson indices, exhibited no significant differences among the four groups (*p* > 0.05) (Figure 4C–F). In contrast, beta diversity analysis revealed a distinct separation of community structure between the control and model groups (Figure 4G). Although partial overlap occurred between the model and two treatment groups, both GP-1 and aspirin interventions shifted the microbial community toward a profile closer to the control group. These results suggested that GP-1 administration could ameliorate AOM/DSS-induced gut dysbiosis.

### 3.9. GP-1 Remodeled the Composition of Gut Flora

**Figure 5:**
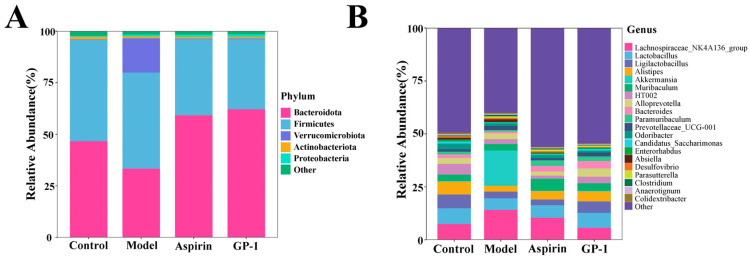
Effect of GP-1 on the composition of gut microbiota in AOM/DSS-treated mice. (**A**) Relative abundance at phylum level; (**B**) relative abundance at genus level (n = 6).

Next, specific changes in the gut microbiota structure were analyzed at different levels. At the phylum level, *Bacteroidota*, *Firmicutes*, *Verrucomicrobiota*, and *Actinobacteriota* were identified as dominant phyla (Figure 5A). Compared with the control group, AOM/DSS treatment severely disrupted the microbiota composition, manifested as a 28.35% reduction in the relative abundance of *Bacteroidota* and a 69.05-fold increase in *Verrucomicrobiota*. As a dominant saccharolytic phylum in the gut, *Bacteroidota* plays a key role in complex carbohydrate degradation. In the GP-1 group, the abundance of *Bacteroidota* increased by 85.68% relative to the model group (*p* < 0.05), accompanied by a decreased *Firmicutes*-to-*Bacteroidota* (F/B) ratio, which might be associated with tumor suppression. A dysregulated increase in the F/B ratio has been reported to correlate with colon tumorigenesis [29]. Additionally, the relative abundance of *Verrucomicrobiota* was significantly reduced in aspirin and GP-1 groups (*p* < 0.05), with levels comparable to those in the control group (*p* > 0.05).

At the genus level, several dominant taxa exhibited intervention-dependent changes in relative abundance. As illustrated in Figure 5B, the abundance of *Lachnospiraceae_NK4A136_group* decreased by 58.97% in the GP-1 group relative to the model group. *Lachnospiraceae_NK4A136_group* has been reported to be enriched in DSS-induced mouse models and participate in pro-inflammatory effects [30]. In contrast, GP-1 administration resulted in a 28.92% increase in *Lactobacillus* relative to the model group, a genus that has been reported to exert anti-inflammatory effects by regulating genes related to the NF-κB signaling pathway [31]. Following GP-1 treatment, the level of *Ligilactobacillus* also increased by 69.14% compared with the model group, a shift related to the maintenance of intestinal barrier integrity [32]. Consistent with the findings of Xu et al. [33], AOM/DSS treatment decreased the abundance of *Alistipes*, and this reduction was partially alleviated by GP-1 (from 2.80% to 4.73%). In addition, GP-1 intervention significantly lowered the abundance of *Akkermansia*, which was abnormally elevated in the model group (*p* < 0.05). The excessive abundance of *Akkermansia* has also been observed in another AOM/DSS-induced mouse model [34] and may trigger mucin breakdown and compromise epithelial integrity [35]. Furthermore, the levels of *Turicibacter*, *Romboutsia*, *Flavonifractor*, and *Adlercreutzia* were significantly reduced after GP-1 treatment compared with the model group (*p* < 0.05) (Figure S2A–D). These genera have been linked to colon tumorigenesis in previous studies [36,37,38]. Together, GP-1 administration helped restore intestinal flora balance in AOM/DSS-treated mice by suppressing potentially harmful bacteria and expanding beneficial bacteria.

LEfSe was employed to analyze microbial taxa variations at different taxonomic levels. Under thresholds of LDA > 3.0 and *p* < 0.05, 35 differential ASVs were identified. The control, model, aspirin, and GP-1 groups exhibited 3, 16, 3, and 13 differentially abundant taxa, respectively (Figure S3A,B). *Akkermansiaceae* was the predominant family in the model group, and *Monoglobaceae* and *Bacteroidaceae* dominated the GP-1 group. These findings further indicated that GP-1 treatment positively influenced intestinal ecological balance by modulating microbial composition.

### 3.10. GP-1 Regulated SCFAs Levels in the Intestine

SCFAs, the key metabolites derived from the microbial fermentation of polysaccharides, play a role in modulating intestinal immune responses and barrier integrity [6]. As shown in Figure S4A, the total SCFA level in the model group increased by 42.94% compared with the control group. This increase might be related to alterations in gut microbiota composition (e.g., increased *Akkermansia*) and mucus degradation, which could potentially contribute to exacerbated intestinal inflammation [39]. Notably, GP-1 treatment increased total SCFA content by 54.22% compared with the model group (*p* < 0.05). The trends of total SCFA and acetic acid were consistent (Figure S4B), and the change in acetic acid was similar to observations reported by Meng et al. [40]. No significant differences in propionic acid were found among groups (Figure S4C). Butyric acid exhibited a significant 65.21% increase in the GP-1 group compared with the model mice (*p* < 0.05) (Figure S4D). As the primary energy source for colonocytes, butyric acid is known to enhance barrier integrity and induce tumor cell apoptosis [6]. These results suggested that GP-1 might promote intestinal health by increasing the production of SCFAs, especially butyric acid.

### 3.11. GP-1 Altered Intestinal Metabolism in AOM/DSS-Treated Mice

**Figure 6:**
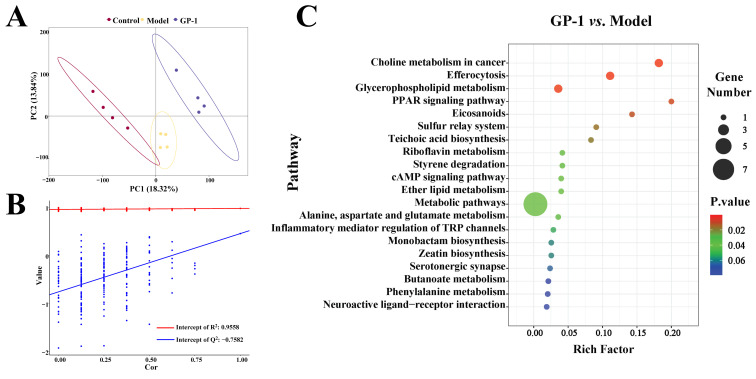
Effect of GP-1 on intestinal metabolites in AOM/DSS-treated mice. (**A**) c score plot; (**B**) permutation test of the PLS-DA model; (**C**) KEGG pathway enrichment analysis of differential metabolites between the GP-1 and model groups (n = 4).

Metabolomic analysis revealed the effects of GP-1 on AOM/DSS-induced intestinal metabolism. As shown in Figure 6A, PLS-DA reflected significant differences in metabolic composition among the three groups, with the model validated by permutation tests (Figure 6B). Compared with the healthy controls, the model group displayed increased levels of 46 metabolites and decreased levels of 60 others (Figure S5A). The downregulated metabolites included glycerol 3-phosphate, cholic acid, deoxycholic acid, and eicosapentaenoic acid (Table S2). GP-1 administration significantly shifted the metabolic profile: 17 metabolites (e.g., lumichrome, uridine) were markedly elevated compared with the model group, while 25 metabolites such as phosphorylcholine and leukotriene B4 were significantly downregulated (Figure S5B and Table S2). These alterations, primarily in lipids, organic acids, and heterocyclic compounds, suggested that GP-1 could modulate the intestinal metabolome in the context of colitis-associated tumors.

KEGG analysis of differential metabolites indicated that the anti-tumor effects of GP-1 involved key pathways such as choline metabolism in cancer, PPAR signaling pathway, eicosanoids, riboflavin metabolism, and cAMP signaling pathway (Figure 6C). Consistent with the enrichment in choline metabolism, GP-1 treatment significantly reduced phosphorylcholine—a key precursor for membrane phospholipid synthesis—and decreased the levels of phosphatidylglycerol (e.g., PG (18:0/18:2 (9Z, 12Z)), PG (16:0/22:5 (4Z, 7Z, 10Z, 13Z, 16Z))) compared with the model group (Table S2). Concurrently, GP-1 also significantly suppressed the levels of leukotriene B4, a pro-inflammatory mediator within eicosanoid biosynthesis, arachidonic acid metabolism, and PPAR signaling pathways [41]. Conversely, lumichrome was significantly increased after GP-1 treatment compared with the model group (Table S2). This metabolite has been reported to trigger tumor cell apoptosis via the p53-dependent pathway [42]. Likewise, GP-1 administration significantly elevated the levels of 3-hydroxybutyric acid and uridine relative to the model group, a change associated with enhanced mucosal barrier repair and reduced pro-inflammatory cytokine expression [43,44]. These findings suggested that the anti-inflammatory and anti-tumor activities of GP-1 might be related to the modulation of potentially pro-tumorigenic and protective metabolites.

### 3.12. GP-1 Modulated Intestinal Gene Expression in AOM/DSS-Treated Mice

**Figure 7:**
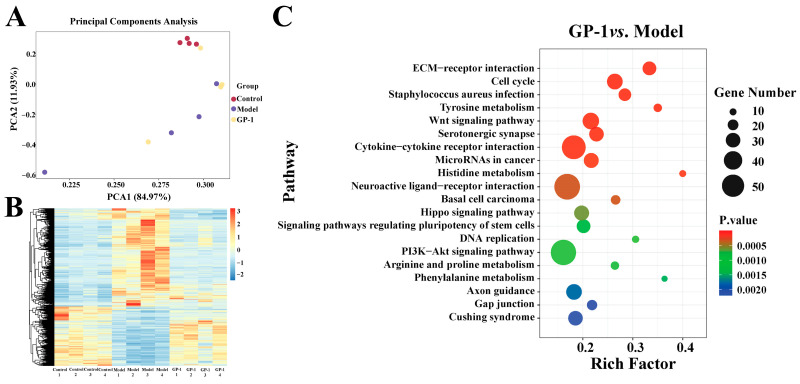
Effect of GP-1 on intestinal gene expression in AOM/DSS-treated mice. (**A**) PCA score plot; (**B**) heatmap of DEGs; (**C**) KEGG pathway enrichment analysis of DEGs between the GP-1 and model groups (n = 4).

To explore the molecular mechanisms underlying the anti-tumor activity of GP-1, colon gene expression was analyzed by RNA-seq. PCA revealed significant differences in gene profiles between control and AOM/DSS-treated mice (Figure 7A). While the GP-1 group partially overlapped with the model group, it tended to converge with the control group, indicating a gene expression pattern similar to controls. A total of 3625 DEGs were identified between the model and control groups (Figure S6A). GP-1 administration significantly altered 2569 genes relative to the model group, comprising 1141 upregulated and 1428 downregulated genes (Figure S6B). A heatmap of these DEGs further suggested that GP-1 treatment partially attenuated the AOM/DSS-induced transcriptional dysregulation (Figure 7B).

Next, GO and KEGG enrichment analyses were conducted on DEGs from the model vs. control and GP-1 vs. model comparisons to investigate the biological functions of GP-1. DEGs from these two datasets were enriched in 1718 and 1284 GO terms respectively, with membrane, cytoplasm, plasma membrane, and protein binding as the predominant GO categories (Figure S6C,D). KEGG analysis further showed that DEGs from both comparisons were significantly enriched in inflammation- and tumor-related pathways (Figures S6E and 7C). Among the top 20 pathways, cytokine–cytokine receptor interaction, PI3K-Akt, Wnt, and ECM-receptor interaction signaling were significantly enriched in both datasets. The coordinated dysregulation of these pathways likely promoted inflammation-driven tumorigenesis, and this aberrant activation was significantly suppressed by GP-1. In the GP-1 group, key genes in cytokine–cytokine receptor interaction (such as *Il1b*, *Tnf*, *Cxcl1*, *Cxcl2*) were markedly downregulated relative to the model group (Table S3). Persistent pro-inflammatory cytokines contribute to the activation of the NF-κB pathway, leading to the upregulation of anti-apoptotic factors (e.g., Bcl-xL) [45]. As a central driver of inflammation-associated tumors, NF-κB can amplify the secretion of TNF-α, IL-1β, and IL-6 and elevate anti-apoptotic and pro-angiogenic factors in colon epithelial cells [45]. Meanwhile, pro-inflammatory cytokines can also trigger the PI3K/Akt cascade, and sustained Akt activation promotes cell survival via cyclin regulation [46]. As shown in Table S3, GP-1 significantly attenuated the overexpression of key genes in the PI3K-Akt and NF-κB pathways (e.g., *Il1b*, *Tnf*, *Il4ra*, *Fgf3*, *Ccne1*, *Jak3*, *Tlr2*) compared with the model group. Wnt signaling also plays an important role in the occurrence of inflammation-induced colon tumors [47]. In this study, GP-1 significantly reduced the expression of *Wnt3*, *Wnt5a*, *Wnt7a*, and *Mmp7* compared with the model group (Table S3). The abnormal upregulation of these Wnt family members can promote tumor cell proliferation by activating cyclin D1, c-Myc, MMP7, and non-canonical Wnt signaling [48,49,50]. In addition, GP-1 intervention effectively attenuated the AOM/DSS-induced hyperactivation of ECM-receptor interaction. The extracellular matrix (ECM) is a major component of the tumor microenvironment, where dysregulated ECM-receptor signaling is conducive to tumor metastasis [51]. Taken together, the inhibitory effect of GP-1 on colitis-associated tumorigenesis might be related to the modulation of key inflammatory and tumorigenic signaling pathways.

### 3.13. Correlation Analysis

To investigate the potential mechanisms of GP-1 in ameliorating colitis-associated colon tumorigenesis, we conducted an integrated correlation analysis of gut microbiota, metabolites, and host genes. As shown in Figure S7A, *Flavonifractor*, *Romboutsia*, *Turicibacter*, and *Akkermansia* were positively correlated with multiple metabolites including leukotriene B4, phosphorylcholine, and phosphatidylglycerols, as well as with inflammatory and tumorigenic genes such as *Il17a*, *Cxcl2*, *Fgf3*, *Wnt5a*, *Nlrp3*, *Tnf*, *Jak3*, *Il1b*, and *Cxcl1* (Figure S7B). In comparison, *Lactobacillus*, *Bacteroides*, *Ligilactobacillus*, and *Alistipes* exhibited opposite correlations, being negatively associated with the aforementioned metabolites but positively correlated with 3-hydroxybutyric acid, uridine, butyric acid, and lumichrome. These results suggested that the gut microbiota and metabolites might play important roles in the protective effects of GP-1 against colitis-associated colon tumorigenesis.

For instance, 3-hydroxybutyric acid was negatively correlated with pro-inflammatory genes such as *Nlrp3*, *Il1b*, and *Tnf* (Figure S7C). Emerging evidence suggests that specific *Lactobacillus* strains can exert hepatoprotective effects by increasing the level of 3-hydroxybutyric acid [52]. Moreover, elevated 3-hydroxybutyric acid can inhibit the activation of NLRP3 inflammasome and downregulate the expression of *Nlrp3*, *Tnf* and *Il1b* [53]. Conversely, leukotriene B4 exhibited positive correlations with genes related to inflammatory responses and tumor proliferation, including *Ccr2*, *Il1b*, *Tnf*, *Cxcl2*, *Il4ra*, *Jak3*, *Tlr2*, *Col1a1*, and *Wnt5a*, while showing negative associations with *Lactobacillus*, *Bacteroides*, and *Alistipes* (Figure S7A–C). In colon tumors, elevated leukotriene B4 may promote inflammation, cell proliferation, and migration by activating PI3K/Akt and NF-κB pathways [54]. *Lactobacillus* has been shown to inhibit leukotriene B4 production [55]. In addition, uridine was negatively correlated with *Tnf*, *Il1b*, and *Cxcl2*, and positively correlated with *Lactobacillus*, *Alistipes*, and *Bacteroides* in this study. It has been reported that uridine can alleviate colitis by reducing IL-6, IL-1β, and TNF [44], and *Lactobacillus plantarum* can increase uridine levels in vivo [56]. Similarly, butyric acid was also positively correlated with *Lactobacillus*, *Bacteroides*, and *Alistipes*, while inversely associated with pro-inflammatory genes (e.g., *Cxcl1*, *Il17a*, and *Il1b*) and proliferation genes (e.g., *Fgf3*, *Wnt3*, and *Jak3*) (Figure S7A–C). Given that *Bacteroides* can degrade polysaccharides to produce SCFAs, and *Lactobacillus*-derived lactic acid can be converted to butyric acid by cross-feeding [57,58], GP-1 might elevate butyric acid levels partly by enriching these bacteria, thereby helping to suppress NF-κB and Wnt signaling [59,60].

Furthermore, *Lactobacillus* exhibits significant anti-inflammatory activity by regulating the NF-κB signaling pathway [31]. Polysaccharide A from *Bacteroides fragilis* has been shown to attenuate colitis via PI3K/Akt modulation [61], and certain *Alistipes* strains can reduce epithelial damage and pro-inflammatory cytokine secretion [62]. These findings indicated that gut microbiota and metabolites are closely related to intestinal inflammation, mucosal homeostasis, and colon tumorigenesis. In conclusion, the alleviating effect of GP-1 on inflammation-driven colon tumorigenesis might be associated with the enrichment of beneficial bacteria, favorable modulation of intestinal metabolic profiles, and reduced activation of tumorigenic signaling pathways.

### 3.14. Structure–Activity Relationships and Potential Anti-Tumor Mechanisms

The above results indicated that GP-1 effectively attenuated colitis-associated tumorigenesis by regulating immune cells, inflammatory factors, oxidative stress, cellular proliferation, intestinal barrier function, gut microbiota, metabolite profiles, and related gene expression. These biological functions of GP-1 might be related to its structural characteristics, including monosaccharide composition, Mw/degree of polymerization, glycosidic bond configuration, branching degree, and conformation. A previous study has shown that prebiotic fructans from various plant sources significantly prevented AOM/DSS-induced intestinal mucosal damage and pro-inflammatory cytokine secretion [63]. Linear (2→1)-linked fructans from *Codonopsis pilosula* effectively reduced pro-inflammatory cytokines and proliferation markers in the same model [64]. Additionally, branched agave fructans exhibited excellent bifidogenic and intestinal barrier-enhancing effects [65]. These observations provide a structural–functional basis for the anti-tumor activity of GP-1.

SCFAs are the main products of the fermentation of indigestible polysaccharides by gut microbiota. In the present study, GP-1 treatment enriched beneficial bacteria, including *Lactobacillus*, *Ligilactobacillus*, and *Bacteroides*, and increased the production of SCFAs (especially butyric acid). *Lactobacillus* primarily produces lactic acid through lactic acid fermentation, and *Bacteroides* produces acetic acid through the acetyl-CoA pathway; both of these can be utilized by other gut microorganisms through cross-feeding to produce butyric acid [58,66]. As signaling molecules, SCFAs can modulate immune responses by activating G protein-coupled receptors and inhibiting histone deacetylases [66]. Among them, butyric acid can inhibit the progression of colitis-associated tumors by suppressing the TLR2/MyD88/NF-κB signaling pathway and the production of IL-6 and TNF-α [67]. Furthermore, butyric acid can inhibit colon tumor cell proliferation and induce apoptosis by modulating Wnt/β-catenin and PI3K/Akt signaling [60,68]. Additionally, other intestinal metabolites revealed by metabolomics and correlation analyses (e.g., 3-hydroxybutyric acid, uridine, and lumichrome) might also contribute to GP-1-mediated modulation of inflammatory and tumor-associated signaling pathways. Collectively, these findings suggested that the inhibitory effects of GP-1 on colitis-associated tumorigenesis might be associated with the remodeling of gut microbiota composition and metabolism.

Nevertheless, several limitations of the present study should be acknowledged. First, the current findings are mainly based on phenotypic observations and multi-omics correlations, and direct causal evidence between the gut microbiota/metabolites and the anti-tumor effects of GP-1 remains to be further validated by approaches such as microbiota transplantation, metabolite supplementation, or gene knockout models. Second, all results were obtained in a mouse model, and the efficacy, safety, dosage regimen, and gut metabolic characteristics of GP-1 in humans still require further clinical investigation. Despite these limitations, this study suggests that GP-1 can serve as a promising functional food ingredient candidate for the prevention of colitis-associated colon tumorigenesis. The regulatory effects of GP-1 on gut microbiota and metabolites highlight its translational potential as a safe natural polysaccharide in intestinal health management and colon tumor prevention.

## 4. Conclusions

This study demonstrated that GP-1, characterized by a (2→1)-linked *β*-d-Fruf backbone with (2→6)-linked *β*-d-Fruf branches, provided significant protection against colitis-associated tumorigenesis in an AOM/DSS mouse model. GP-1 treatment effectively ameliorated disease pathology by restoring intestinal barrier integrity, suppressing excessive cell proliferation, and attenuating gut inflammation. Furthermore, GP-1 remodeled the gut microbiota composition, enriching beneficial bacteria (e.g., *Lactobacillus* and *Bacteroides*) while reducing potentially harmful bacteria, and increased the production of SCFAs and alleviating intestinal metabolic dysregulation. Transcriptomic analysis indicated that GP-1 significantly suppressed the hyperactivation of pro-inflammatory and pro-tumorigenic signaling pathways in colon tissue. Collectively, our findings delineated the chemopreventive potential of GP-1 against colitis-associated colon tumors and elucidated the underlying mechanisms involving the modulation of gut microbiota, metabolites, and critical inflammatory/tumorigenic signaling pathways.

## Data Availability

The original contributions presented in the study are included in the article/Supplementary Materials, further inquiries can be directed to the corresponding author.

## Supplementary Materials

The following supporting information can be downloaded at https://www.mdpi.com/article/10.3390/foods15111901/s1: Figure S1: Structural characteristics of GP-1; Figure S2: Relative abundance of *Turicibacter* (A); *Romboutsia* (B); *Flavonifractor* (C); and *Adlercreutzia* (D); Figure S3: LEfSe analysis; Figure S4: Effect of GP-1 on fecal SCFA production; Figure S5: Volcano plots of differential metabolites in model vs. control (A) and GP-1 vs. model (B); Figure S6: Transcriptomic analysis of the colon in AOM/DSS-treated mice; Figure S7: Correlation analysis of gut microbiota, metabolites, and DEGs related to inflammation and tumors; Table S1: DAI scoring criteria; Table S2: Fold changes of significantly altered metabolites across comparison groups; Table S3: Fold changes of representative DEGs in inflammation- and tumor-associated KEGG pathways across comparison groups.

## Abbreviations

The following abbreviations are used in this manuscript:

AB-PAS	Alcian blue-periodic acid-Schiff
ANOVA	Analysis of variance
AOD	Average optical density
AOM	Azoxymethane
ASVs	Amplicon sequence variants
CAT	Catalase
CD3^+^	Cluster of differentiation 3^+^
CD68^+^	Cluster of differentiation 68^+^
COX-2	Cyclooxygenase-2
CXCL1	C-X-C motif chemokine ligand 1
DAI	Disease activity index
DEG	Differentially expressed gene
DSS	Dextran sulfate sodium
FTIR	Fourier transform infrared
GO	Gene Ontology
H&E	Hematoxylin and eosin
IFN-γ	Interferon-γ
IL-6	Interleukin-6
IL-10	Interleukin-10
iNOS	Inducible nitric oxide synthase
KEGG	Kyoto Encyclopedia of Genes and Genomes
MCP-1	Monocyte chemoattractant protein-1
MDA	Malondialdehyde
MPO	Myeloperoxidase
Mw	Molecular weight
PCA	Principal component analysis
PLS-DA	Partial least squares discriminat analysis
SCFA	Short-chain fatty acid
SOD	Superoxide dismutase
TNF-α	Tumor necrosis factor-α
UV–Vis	Ultraviolet–visible
