# Supplementary material for: Garlic Polysaccharides Ameliorate AOM/DSS-Induced Colon Tumorigenesis: A Multi-Omics Mechanistic Study
Source: Foods. 2026 May 28;15(11):1901. doi: 10.3390/foods15111901 (PMC13256388; doi:10.3390/foods15111901)
Supplement: Supplementary file 1 [file foods-15-01901-s001.zip › foods-4204211-supplementary.pdf]

Supporting Information

Garlic polysaccharides ameliorate AOM/DSS-induced colon tumorigenesis: A multi-omics mechanistic study

Yongqiu Qi [a], Xiaoming Lu [a], Lingyu Li [a], Zhenjia Zheng [a], Yiteng Qiao [a, *]

[a] Key Laboratory of Food Nutrition and Health in Universities of Shandong, College of Food Science and Engineering, Shandong Agricultural University, 61 Daizong Street, Tai'an, Shandong 271018, P.R. China

*Corresponding authors: Yiteng Qiao (Email: yitengqiao@sdau.edu.cn).

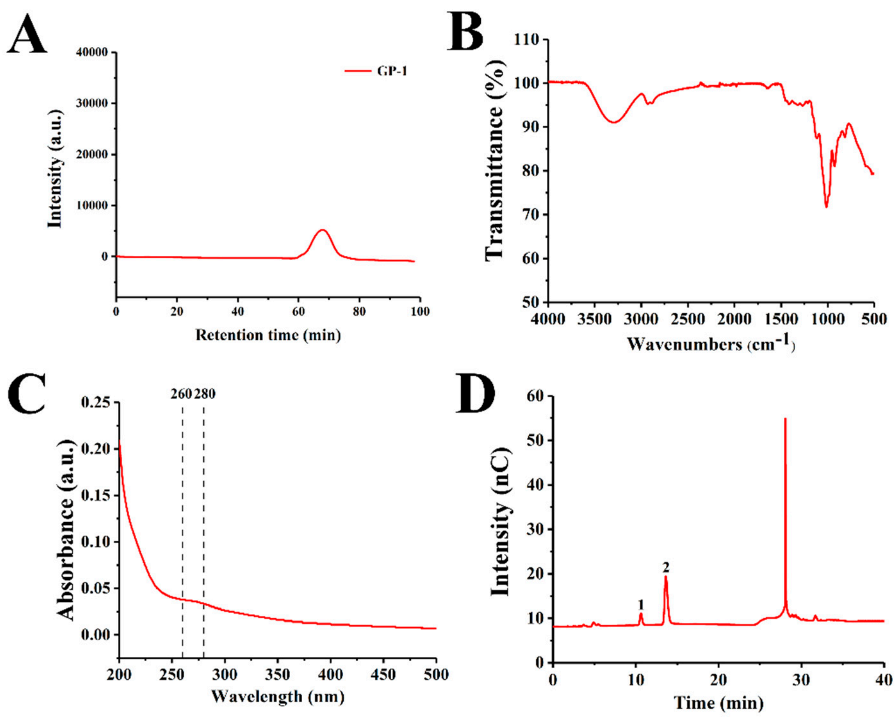

Figure S1. Structural characteristics of GP-1. (A) Mw; (B) FTIR spectrum; (C) UV-vis spectrum; (D) Monosaccharide profile. (1) Glucose, (2) Fructose.

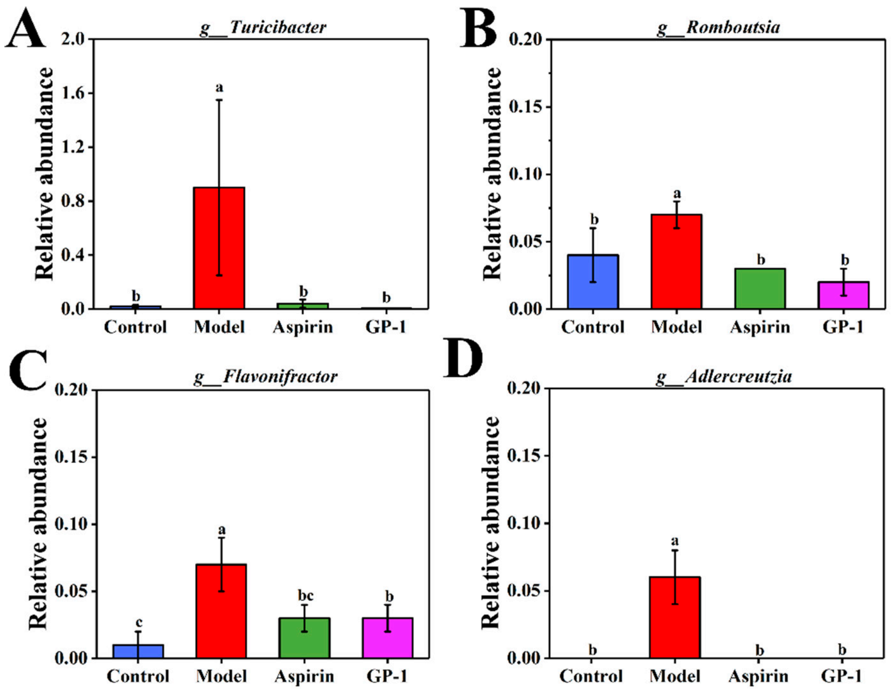

Figure S2. Relative abundance of *Turicibacter* (A); *Romboutsia* (B); *Flavonifractor* (C); and *Adlercreutzia* (D). The data are shown as mean ± SD. Different letters mean significant differences ($P < 0.05$).

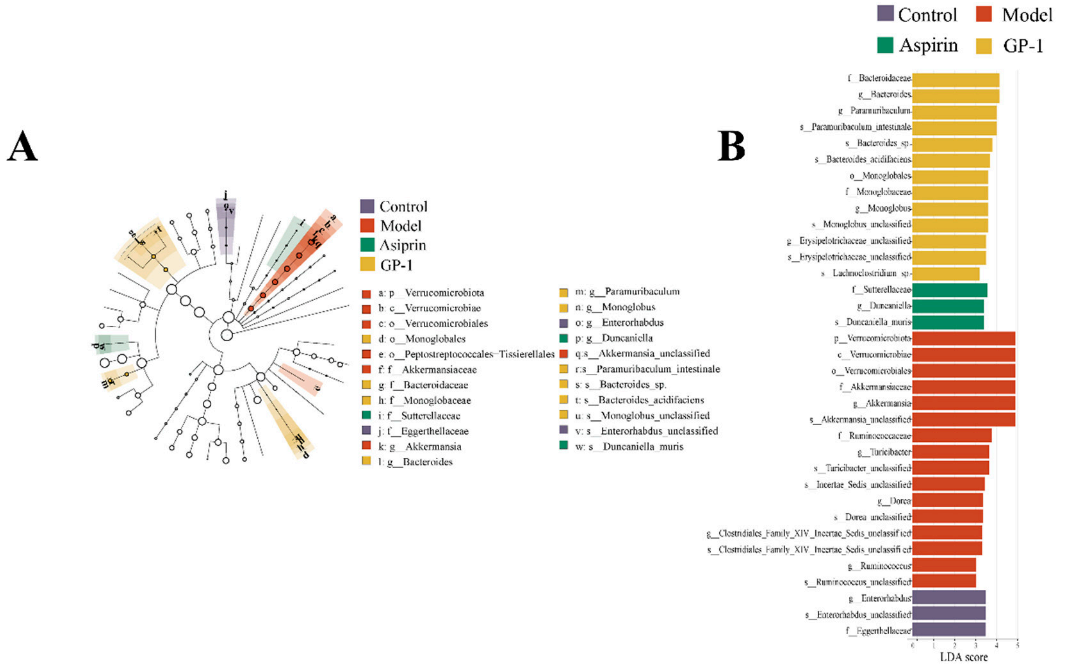

Figure S3. LEfSe analysis. (A) Cladogram; (B) Bar plot of LDA scores.

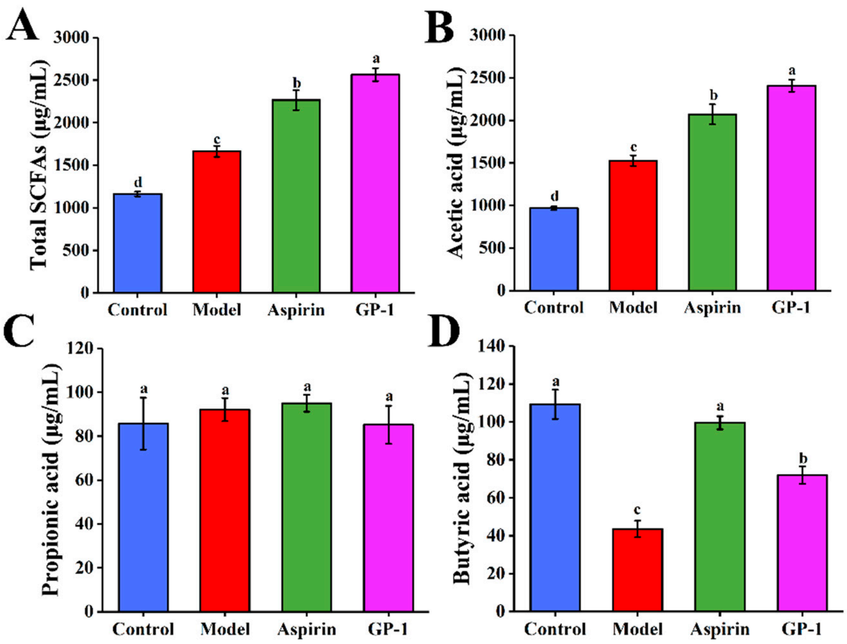

Figure S4. Effect of GP-1 on fecal SCFA production. (A) Total SCFAs; (B) Acetic acid; (C) Propionic acid; (D) Butyric acid. The data are shown as mean ± SD. Different letters mean significant differences ($P < 0.05$).

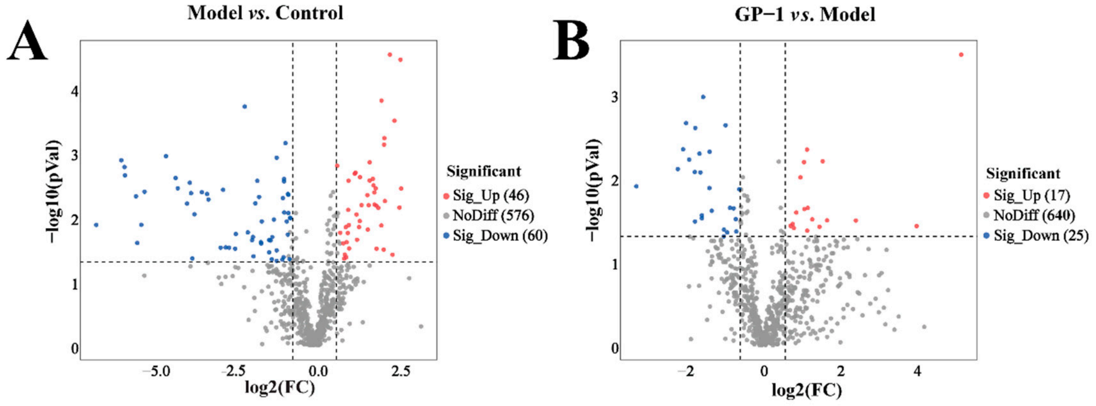

Figure S5. Volcano plots of differential metabolites in model *vs*. control (A) and GP-1 *vs*. model (B).

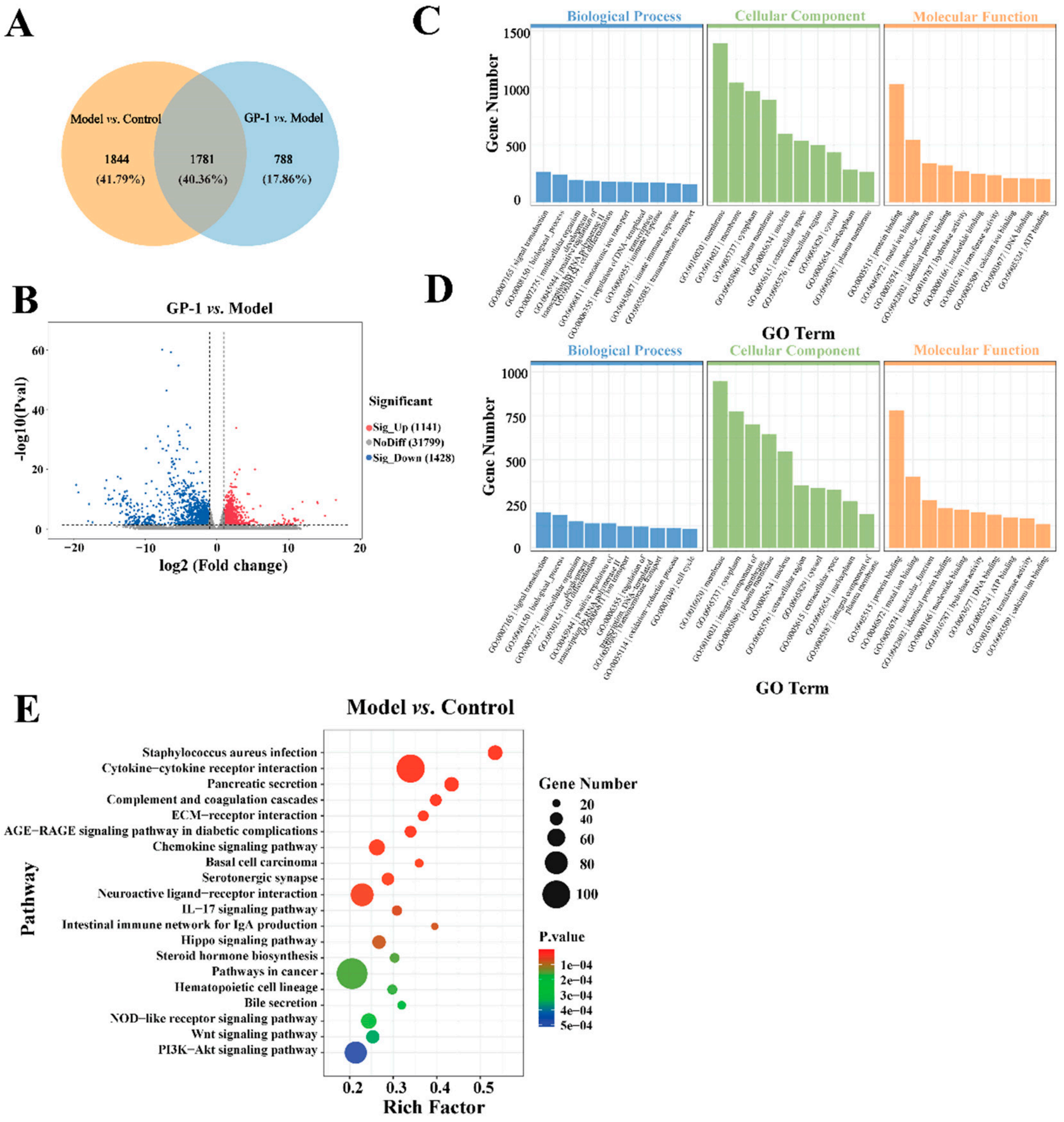

Figure S6. Transcriptomic analysis of the colon in AOM/DSS-treated mice. (A) Venn diagram of DEGs among groups; (B) Volcano plot of DEGs in GP-1 *vs*. model; (C) GO enrichment analysis of DEGs in model *vs*. control; (D) GO enrichment analysis of DEGs in GP-1 *vs*. model; (E) KEGG enrichment analysis of DEGs in model *vs*. control (n = 4).

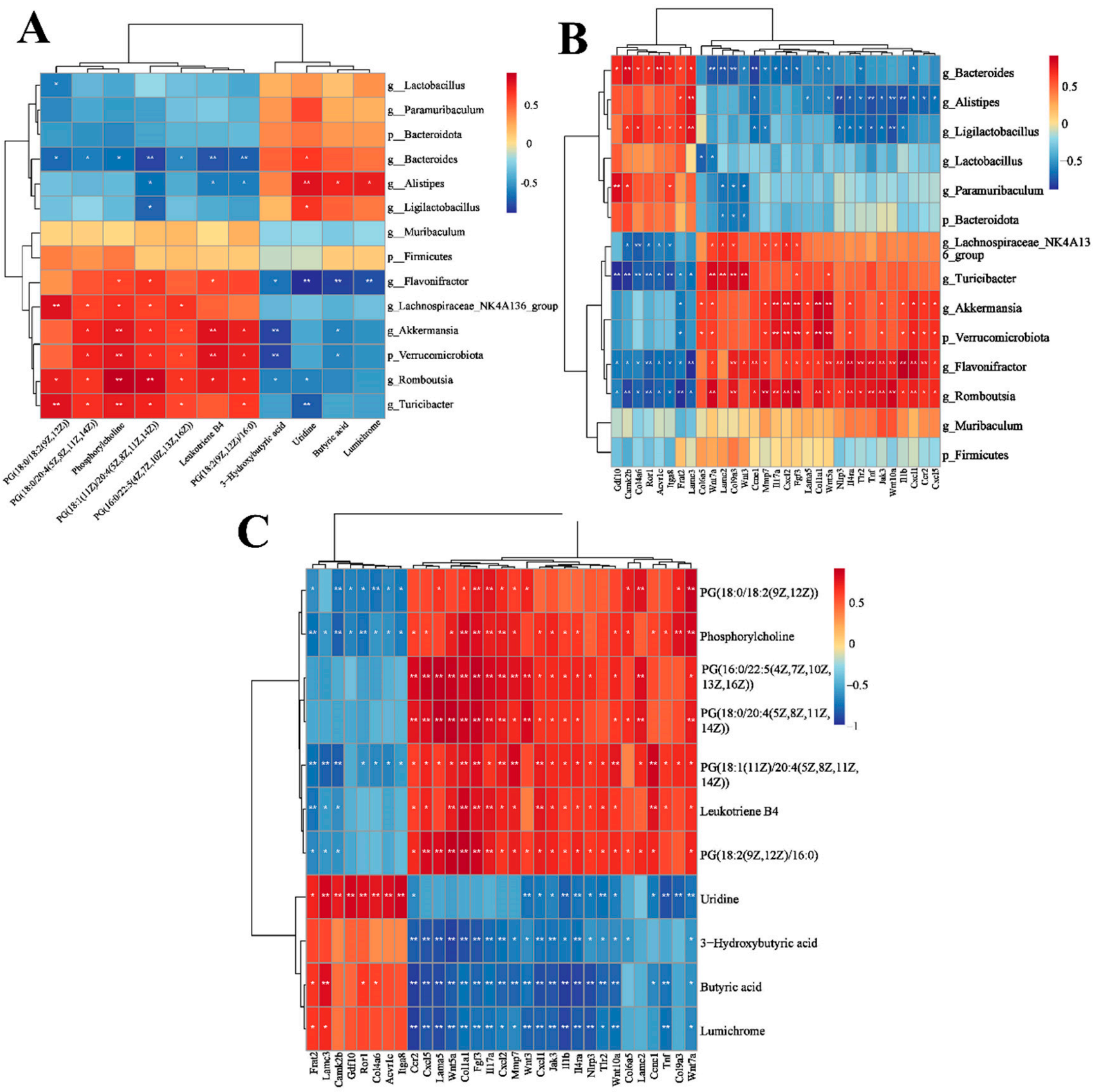

Figure S7. Correlation analysis of gut microbiota, metabolites, and DEGs related to inflammation and tumors. (A) Spearman correlation of key gut microbiota and metabolites; (B) Spearman correlation of gut microbiota and DEGs; (C) Spearman correlation of key metabolites and DEGs.

Table S1 DAI scoring criteria.

Parameter	Weight loss (D1)	Stool consistency (D2)	Intestinal bleeding (D3)
Score 0	0	Normal	No blood
Score 1	1%-5%	Soft but formed stool	Slight bleeding
Score 2	5-10%	Loose stool (no diarrhea)	Visible blood in stool
Score 3	10-20%	Mild diarrhea	--
Score 4	>20%	Severe diarrhea	Gross rectal bleeding

Note: DAI=(D1+D2+D3)/3

Table S2 Fold changes of significantly altered metabolites across comparison groups.

Number	Metabolites	Model vs Control (FC)	GP-1 vs Model (FC)
1	Glycerol 3-phosphate	0.26 ↓	
2	Niacinamide	1.89 ↑	
3	Cholic acid	0.06 ↓	
4	Butyrylcarnitine	0.57 ↓	
5	3-Hydroxybutyric acid	0.32 ↓	1.83 ↑
6	L-Acetylcarnitine	3.13 ↑	
7	DL-Acetylcarnitine	2.67 ↑	
8	1-Pyrrolidinecarboxaldehyde	2.38 ↑	
9	LysoPC(16:1/0:0)	1.98 ↑	
10	1,2-Didecanoyl-sn-glycero-3-phosphocholine	0.02 ↓	
11	3-hydroxyoctanoyl carnitine	3.74 ↑	
12	Sphinganine	0.41 ↓	
13	LysoPC(18:2/0:0)	0.31 ↓	
14	Daidzein	0.02 ↓	
15	Riboprine	0.58 ↓	
16	Phenol sulphate	0.13 ↓	5.38 ↑
17	Uridine	0.59 ↓	1.98 ↑
18	Deoxycholic acid	0.10 ↓	
19	O(6)-Benzylguanine	0.52 ↓	
20	3-Hydroxyhexadecanoylcarnitine	4.45 ↑	
21	Eicosapentaenoic acid	0.26 ↓	
22	Dihydroxyacetone phosphate	3.64 ↑	
23	N-pyrimidin-5-ylformamide	0.27 ↓	
24	7,8-Dihydro-3-methylpyrrolo[1,2-a]pyrimidin-2(6H)-one	0.07 ↓	
25	3-Oxocholic acid	0.04 ↓	
26	Hexanoylcarnitine	1.86 ↑	
27	Hydroquinone sulfate	0.05 ↓	
28	Dihydroferulic acid	0.22 ↓	
29	Tiglylcarnitine	2.08 ↑	0.51 ↓
30	Dodecanedioic acid	0.28 ↓	
31	12-KETE	2.11 ↑	
32	Pyrocatechol sulfate	0.02 ↓	
33	3-hydroxydecanoyl carnitine	5.10 ↑	
34	Prostaglandin H2	0.55 ↓	
35	9-Decenoylcarnitine	3.96 ↑	
36	2-Hydroxymyristoylcarnitine	4.23 ↑	
37	17,18-DiHETE	1.96 ↑	
38	Heneicosapentaenoic acid	0.44 ↓	
39	12-Hydroxystearic acid	0.18 ↓	

40	PG (18:0/20:4(5Z,8Z,11Z,14Z))	3.52 ↑	0.38 ↓
41	3-Hydroxy-11Z-octadecenoylcarnitine	3.81 ↑	
42	4-Hydroxy-2-butenoic acid gamma-lactone	0.37 ↓	
43	3-Hydroxy-cis-5-tetradecenoylcarnitine	3.15 ↑	
44	glyco-beta-muricholic acid	0.40 ↓	
45	13-Hydroxymarasmene	0.16 ↓	
46	1,2-Dihexanoyl-sn-glycero-3-phosphoethanolamine	0.05 ↓	
47	PI(18:1(11Z)/16:0)	1.92 ↑	
48	Leukotriene B4	3.79 ↑	0.29 ↓
49	D-Ribose 5-phosphate	1.62 ↑	
50	PG(18:3(9Z,12Z,15Z)/18:1(9Z))	3.28 ↑	
51	Stearoylethanolamide	0.56 ↓	
52	3-(3-Hydroxyphenyl)propanoic acid	0.44 ↓	
53	Quercetin 3-(6''-malonyl-glucoside)	0.52 ↓	
54	5-Methyl-2-furancarboxaldehyde	0.15 ↓	
55	1,2-Dioleoyl-sn-glycero-3-phosphoglycerol	5.62 ↑	0.24 ↓
56	LysoPI(20:4(5Z,8Z,11Z,14Z)/0:0)	0.53 ↓	
57	3-hydroxydodecanoyl carnitine	4.49 ↑	
58	PG(18:1(9Z)/22:6(4Z,7Z,10Z,13Z,16Z,19Z))	2.42 ↑	0.49 ↓
59	LysoPI(16:0/0:0)	0.52 ↓	2.23 ↑
60	1-[(2R,3S,5R)-3,4-Dihydroxy-5-(hydroxymethyl)oxolan-2-yl]pyrimidine-2,4-dione	0.50 ↓	2.11 ↑
61	Isobutyryl-L-carnitine	0.56 ↓	
62	2-(Acetylamino)-2-deoxy-alpha-D-mannopyranose	2.75 ↑	
63	N8-Acetylspermidine	2.07 ↑	
64	PG(18:0/18:2(9Z,12Z))	2.61 ↑	0.32 ↓
65	5-Cholesten-3beta-25-diol-3-sulfate	0.09 ↓	
66	Lumichrome	0.24 ↓	1.72 ↑
67	Dimethylmyleran	0.39 ↓	2.26 ↑
68	PG(18:1(11Z)/18:2(9Z,12Z))	4.52 ↑	0.29 ↓
69	SM(d18:1/18:1)	5.39 ↑	0.10 ↓
70	3-[3-(Sulfooxy)phenyl]propanoic acid	0.10 ↓	
71	PI(16:0/16:1(9Z))	4.57 ↑	
72	Homomethionine	0.29 ↓	
73	PG(16:0/22:5(4Z,7Z,10Z,13Z,16Z))	4.24 ↑	0.38 ↓
74	Leucylalanine	2.50 ↑	
75	Prostaglandin J2	2.05 ↑	
76	Cadabicilone	0.07 ↓	
77	Hovenine A	0.07 ↓	
78	PS(18:1(9Z)/18:0)	0.40 ↓	
79	6-beta-hydroxymedroxyprogesterone	3.57 ↑	
80	1-Stearoylglycerophosphoglycerol	0.50 ↓	2.45 ↑
81	Docosapentaenoic acid (22n-3)	0.51 ↓	
82	PG(18:1(11Z)/20:4(5Z,8Z,11Z,14Z))	2.65 ↑	0.32 ↓

83	Bis(2-ethylhexyl) phthalate	0.18 ↓	
84	17-Hydroxyandrostane-3-glucuronide	0.07 ↓	
85	PG(18:0/22:5(4Z,7Z,10Z,13Z,16Z))	6.40 ↑	0.26 ↓
86	FA 18:5+2O	0.01 ↓	16.15 ↑
87	Enterolactone	0.02 ↓	
88	Monoisononyl phthalate	0.02 ↓	
89	PG(18:1(11Z)/22:4(7Z,10Z,13Z,16Z))	6.50 ↑	
90	12S-HHT	0.57 ↓	
91	PG(16:0/22:4(7Z,10Z,13Z,16Z))	6.25 ↑	0.34 ↓
92	N-alpha-Acetyl-L-lysine	2.47 ↑	
93	PG(18:2(9Z,12Z)/16:0)	3.29 ↑	0.29 ↓
94	xi-10-Hydroxyoctadecanoic acid	0.32 ↓	
95	7C-aglycone	0.02 ↓	
96	7,8-Dihydropteroic acid	0.03 ↓	
97	Isoleucyl-Asparagine	0.44 ↓	
98	12,13-DiHODE	0.37 ↓	
99	15-Deoxy-d-12,14-PGJ2	2.10 ↑	
100	2-Phenylpropionaldehyde dimethyl acetal	0.59 ↓	
101	10-alpha-methoxy-9,10-dihydrolysergol	0.14 ↓	
102	2-beta-hydroxymedroxyprogesterone	3.65 ↑	
103	2-Hydroxydecanedioic acid	0.30 ↓	
104	PG(16:1(9Z)/22:5(4Z,7Z,10Z,13Z,16Z))	3.21 ↑	0.53 ↓
105	Aminoadipic acid	1.74 ↑	
106	PG(18:3(6Z,9Z,12Z)/22:5(4Z,7Z,10Z,13Z,16Z))	2.00 ↑	0.50 ↓
107	Phosphorylcholine		0.55 ↓
108	LysoPA(P-16:0/0:0)		1.76 ↑
109	D-Aspartic acid		0.59 ↓
110	PG(16:0/20:4(5Z,8Z,11Z,14Z))		0.25 ↓
111	S-Adenosylmethionine		0.62 ↓
112	FA 22:5		2.96 ↑
113	Ethenodeoxyadenosine		0.65 ↓
114	2,3-Butanedithiol		0.33 ↓
115	17-HDoHE		1.65 ↑
116	LysoPA(0:0/18:0)		2.79 ↑
117	Methyl (2E)-2-(10,13-dimethyl-11-oxo-3-pyrrolidin-1-yl-2,7,8,9,12,14,15,16-octahydro-1H-cyclopenta[a]phenanthren-17-ylidene) acetate		2.23 ↑
118	DG (16:0/18:1(9Z)/0:0)		0.40 ↓
119	LysoPC(0:0/18:1(9Z))		2.13 ↑
120	D-Kynurenine		0.33 ↓
121	Aspartylphenylalanine		36.19 ↑
122	LysoPC(P-18:1(9Z)/0:0)		3.20 ↑
123	LysoPC(18:4(6Z,9Z,12Z,15Z)/0:0)		0.22 ↓
124	Phenylacetic acid		0.61 ↓

Table S3 Fold changes of representative DEGs in inflammation- and tumor-associated KEGG pathways across comparison groups.

Number	Gene name	Pathway name	Model *vs.* Control (FC)	GP-1 *vs.* Model (FC)
1	Cxcl1	Cytokine-cytokine receptor interaction	8.69 ↑	0.17 ↓
2	Ccr2	Cytokine-cytokine receptor interaction	5.18 ↑	0.49 ↓
3	Acvr1c	Cytokine-cytokine receptor interaction	0.33 ↓	3.86 ↑
4	Gdf10	Cytokine-cytokine receptor interaction	0.44 ↓	3.46 ↑
5	Cxcl5	Cytokine-cytokine receptor interaction	362.22 ↑	0.02 ↓
6	Il17a	Cytokine-cytokine receptor interaction	79.97 ↑	0.08 ↓
7	Il1b	Cytokine-cytokine receptor interaction, NF-κB signaling pathway	19.66 ↑	0.08 ↓
8	Tnf	Cytokine-cytokine receptor interaction, NF-κB signaling pathway	3.33 ↑	0.32 ↓
9	Cxcl2	Cytokine-cytokine receptor interaction, NF-κB signaling pathway	312.68 ↑	0.02 ↓
10	Il4ra	Cytokine-cytokine receptor interaction, PI3K-Akt signaling pathway,	2.73 ↑	0.49 ↓
11	Fgf3	PI3K-Akt signaling pathway	32548.67 ↑	0.01 ↓
12	Ccne1	PI3K-Akt signaling pathway	2.70 ↑	0.35 ↓
13	Jak3	PI3K-Akt signaling pathway	2.78 ↑	0.47 ↓
14	Tlr2	PI3K-Akt signaling pathway		0.46 ↓
15	Pik3r6	PI3K-Akt signaling pathway	2.12↑	
16	Pik3cg	PI3K-Akt signaling pathway	2.35↑	
17	Pik3ap1	PI3K-Akt signaling pathway	3.06↑	0.49↓
15	Col9a3	PI3K-Akt signaling pathway, ECM-receptor interaction	40.48 ↑	0.02 ↓
16	Col4a6	PI3K-Akt signaling pathway, ECM-receptor interaction	0.16 ↓	6.64 ↑

17	Col6a5	PI3K-Akt signaling pathway, ECM-receptor interaction		0.37 ↓
18	Col1a1	PI3K-Akt signaling pathway, ECM-receptor interaction	3.18 ↑	0.41 ↓
19	Lamc2	PI3K-Akt signaling pathway, ECM-receptor interaction		0.33 ↓
20	Lama5	PI3K-Akt signaling pathway, ECM-receptor interaction	3.24 ↑	0.39 ↓
21	Itga8	PI3K-Akt signaling pathway, ECM-receptor interaction	0.44 ↓	3.40 ↑
22	Lamc3	PI3K-Akt signaling pathway, ECM-receptor interaction	0.19 ↓	5.28 ↑
23	Mmp7	Wnt signaling pathway	2540.09 ↑	0.00 ↓
24	Wnt7a	Wnt signaling pathway	46.68 ↑	0.00 ↓
25	Wnt3	Wnt signaling pathway	19.93 ↑	0.05 ↓
26	Camk2b	Wnt signaling pathway		2.76 ↑
27	Ror1	Wnt signaling pathway	0.43 ↓	2.70 ↑
28	Frat2	Wnt signaling pathway	0.39 ↓	2.62 ↑
29	Wnt5a	Wnt signaling pathway	3.24 ↑	0.38 ↓
30	Wnt10a	Wnt signaling pathway	145.66 ↑	0.01 ↓
31	Nlrp3	NOD-like receptor signaling pathway	7.42 ↑	0.23 ↓
